# Routine data from prevention of mother-to-child transmission (PMTCT) HIV testing not yet ready for HIV surveillance in Mozambique: a retrospective analysis of matched test results

**DOI:** 10.1186/1471-2334-13-96

**Published:** 2013-02-22

**Authors:** Peter W Young, Mussagy Mahomed, Roberta Z Horth, Ray W Shiraishi, Ilesh V Jani

**Affiliations:** 1 Division of Global HIV/AIDS, Centers for Disease Control and Prevention (CDC), Maputo, Mozambique, 193 Av Kenneth Kaunda, P.O. Box 783, 10002, Maputo, Mozambique; 2 Instituto Nacional de Saúde, Maputo, Mozambique; 3 University of California San Francisco, Maputo, Mozambique; 4 Division of Global HIV/AIDS, Centers for Disease Control and Prevention (CDC), Atlanta, GA, USA

**Keywords:** HIV prevalence, HIV program data, HIV surveillance, Prevention of mother-to-child transmission, HIV diagnostic tests, Sentinel surveillance, Mozambique

## Abstract

**Background:**

Opt-out HIV testing is offered at 70% of antenatal care (ANC) clinics in Mozambique through the prevention of mother-to-child transmission (PMTCT) program. If routine data from this program were of sufficient quality, their heightened coverage and continuous availability could complement or even replace biannual sentinel serosurveys that currently serve as the primary HIV surveillance system in Mozambique.

**Methods:**

We assessed the efficacy of routine HIV testing data from prevention of mother-to-child transmission programs for estimating the prevalence of HIV infection among pregnant women. The PMTCT program uses sequential point-of-care rapid tests conducted on site while ANC surveillance surveys use dried blood spots tested sequentially for HIV-1/2 antibodies at a central laboratory. We compared matched routine PMTCT and ANC surveillance test results collected during 2007 and 2009 ANC surveillance surveys from 36 sentinel sites.

**Results:**

After excluding 659 women without PMTCT data, including 83 who refused rapid testing, test results from a total of 20,563 women were available. Pooling the data from both years indicated HIV prevalence from routine PMTCT testing was 14.4% versus 15.2% from surveillance testing (relative difference -5.1%; absolute difference -0.78%). Positive percent agreement (PPA) of PMTCT versus surveillance tests was 88.5% (95% Confidence Interval [CI]: 85.7-91.3%), with 19 sites having PPA below 90%; Negative percent agreement (NPA) was 98.9% (CI: 98.5-99.2%). No significant difference was found among three regions (North, Center and South), however both PPA and NPA were significantly higher in 2009 than 2007 (p < 0.05).

**Conclusions:**

We found low PPA of PMTCT test results compared to surveillance data which is indicative either of testing errors or data reporting problems. Nonetheless, PPA improved significantly from 2007 to 2009, a possible positive trend that should be investigated further. Although use of PMTCT test results would not dramatically change HIV prevalence estimates among pregnant women, the impact of site-level differences on surveillance models should be evaluated before these data are used to replace or complement ANC surveillance surveys.

## Background

Mozambique has a generalized HIV epidemic with adult prevalence estimated at 11.5% [1]. Surveillance data show the prevalence stabilizing at the national level, but with distinct regional levels and trends [2].

Mozambique began using serosurveys in ANC clinics for HIV surveillance in 1988. Antenatal care HIV surveillance was restricted to one site in the capital city until the end of the civil war in 1992, but expanded rapidly in the late nineties, reaching 36 sites located in both urban and rural areas and in all 11 provinces by 2001. These surveys are currently conducted every two years and as of 2007 have been based on centralized Enzyme-Linked Immunosorbent Assay (ELISA) testing of dried blood spot (DBS) specimens for presence of HIV antibodies prepared from leftover blood from routine syphilis tests. The national health service began offering rapid HIV tests at ANC clinics as part of the Mozambican national prevention of mother-to-child transmission (PMTCT) program in 2003. With the expansion of HIV testing in PMTCT program settings in Mozambique, 70% of all ANC sites in Mozambique provided opt-out rapid HIV testing with same day return of results during ANC visits; as of 2007 all 36 sentinel surveillance sites have provided this service. Women who attend ANC are generally representative of all pregnant women in Mozambique due to high (86%) ANC coverage [3]. If routine HIV test data from the PMTCT program can provide reliable estimates of HIV prevalence in pregnant women, these data could supplement or replace traditional ANC serosurveys, at lower cost and potentially with greater frequency, coverage and precision.

Before relying on such data for surveillance purposes, it is first necessary to evaluate: 1) the availability of routine PMTCT program data; 2) differences in test results between the PMTCT and ANC surveillance programs; and 3) any bias that may be introduced by differences in uptake, data quality, and testing procedures between these data sources. In this paper we examine differences in test results and test uptake between the PMTCT and ANC surveillance programs in 2007 and 2009.

There are well-established procedures for evaluating new diagnostic methods against a gold standard diagnostic method and reporting the results from such evaluations [4,5]. We felt that in this study discrepant results could be due to differences in viral detection due to differing window periods, errors in rapid testing, centralized ELISA testing, or specimen handling, or a combination of reasons. Therefore, we treated centralized ELISAs as a non-reference standard, as has been recommended in the case of evaluating a diagnostic system when a gold standard is not available [6].

We used existing data collected through the HIV surveillance system to evaluate the reliability of PMTCT and ANC surveillance test results and to identify potential biases due to refusals and other causes of missing HIV test results. Based on this, we further consider the potential impact on HIV prevalence estimates if routine PMTCT data were used instead of traditional surveillance data from ANC surveys. Since the surveillance system itself was used to collect the data reported in this evaluation, questions of availability and quality of routine PMTCT data in Mozambique, though important considerations, are not evaluated here, but are being assessed through a separate ongoing study.

## Methods

ANC surveillance procedures in Mozambique are based on UNAIDS/WHO guidelines [7]. In 2007 Mozambique adopted an informed consent process for ANC surveillance. After participation in a group discussion with the ANC nurse, during their first ANC visit women are asked for verbal informed consent for use of leftover blood from syphilis testing to prepare DBS specimens with linked demographic data for HIV surveillance purposes. Those who refuse to give blood for syphilis testing are ineligible for the survey, although those refusing syphilis testing or ANC surveillance could still participate in PMTCT HIV testing. Similarly, those who refuse a PMTCT HIV test are still encouraged to participate in ANC surveillance. ANC surveillance testing is anonymous and surveillance test results are not returned to participants. Although women had the opportunity to refuse ANC surveillance, uptake was 98.9% in both 2007 and 2009 [2,8].

The PMTCT program is integrated into ANC in Mozambique. Provider-initiated testing and counseling is performed by the maternal-child health nurse during the first ANC visit and is based on the national HIV diagnostic testing algorithm [4,9]. Women are first screened with Determine HIV-1/2 (Abbott Laboratories, Abbott Park, Illinois, USA, sensitivity 100.0% [CI: 95.5-100.0%], specificity 99.4% [CI: 96.7-100.0%] [10]); those that test positive are confirmed with Uni-Gold *HIV* (Trinity Biotech, Dublin, Ireland, sensitivity 100.0% [CI: 95.5-100.0%], specificity 100.0% [CI: 97.9-100.0%] [10]); discordant results are considered indeterminate and clients are asked to return after one month for repeat testing. During the surveillance round a single venous blood draw using vacuum tubes is used to prepare rapid tests, a rapid plasma reagin (RPR) syphilis test, and for those who consent to participate, DBS specimens for centralized ANC surveillance testing.

During the ANC surveillance survey, nurses documented in the ANC surveillance register whether the client consented to participate in ANC surveillance, whether the client had an HIV test conducted as part of the PMTCT program, and whether the client had ever had a previous HIV positive test result. Results from rapid HIV tests conducted by the PMTCT program were recorded along with demographic data and a unique code. If the client consented to ANC surveillance, the nurse also labeled filter paper cards with the same unique code and prepared DBS specimens which were later sent to the National Immunology Reference Lab in Maputo for centralized antibody testing with ELISA. ELISA testing included a Vironostika HIV Uniform II plus O (bioMérieux bv, Netherlands, sensitivity 100.0% [CI: 99.6-100.0%], specificity 100.0% [CI: 99.7-100.0%]) screening test followed by confirmation of positive specimens with Murex HIV 1-2-O (Abbott Laboratories, UK, sensitivity 100% [CI: 99.97-100%], specificity 99.91% [CI: 99.82-99.96%]). Discordant results were considered HIV-negative. As results from repeat testing of women with indeterminate results were not available for analysis, in this analysis indeterminate PMTCT rapid test results were recoded as HIV-negative for comparison with ELISA test results for consistency.

Records were limited to the first 300 ANC surveillance participants per sentinel site per year to ensure similar weighting across sites. Test results were matched by unique code, pooled across the 36 surveillance sites and surveys from the two years (2007 and 2009) included in this analysis, and then disaggregated by region and year for further analysis. It was not possible to remove women who may have participated in both the 2007 and 2009 surveys. Although pooled prevalence was used for this study, the median of the site-level HIV prevalence is also reported for consistency with WHO recommendations [7].

Data were analyzed retrospectively to assess consistency of test results between routine PMTCT and ANC surveillance data, with the surveillance algorithm using second generation antibody tests and no tie-breaker for resolving indeterminate results. Thus, the final ELISA result serves as a non-reference standard which may itself, in some cases, produce an erroneous diagnosis. In such cases, the matched rapid test results may either also be false positive or false negative (leading to high agreement but low sensitivity and specificity) or may represent the correct diagnosis (leading to lower agreement but high sensitivity and specificity). Positive percent agreement (PPA) and negative percent agreement (NPA) were calculated to assess the level of agreement between the rapid test results under evaluation and the non-reference standard ELISA result. The prevalence ratio between refusers and non-refusers of PMTCT testing and the percent bias due to refusals that would be introduced if ANC surveillance were based on routine PMTCT tests instead of ELISA test results were also calculated.

Analyses were performed with R Version 2.13.1 [11], including analyses of complex samples which were done using the R survey module [12]. Comparisons of PPA and NPA were done using SAS Software Version 9.2 (SAS Institute Inc, Cary, NC) using the Rao-Scott chi-square test implemented in PROC SURVEYFREQ. Each surveillance site was treated as a separate cluster and all women were given equal weight. Results with a p-value < 0.05 were considered significant.

The ANC surveillance protocol, including the comparison of ANC surveillance data with routine PMTCT test results, was approved by the Mozambican Bioethics Committee and the U.S. Centers for Disease Control and Prevention (CDC).

## Results

After excluding 659 women who did not have PMTCT rapid test results, including 83 due to refusal to have a rapid test performed, 20,563 women had HIV rapid and ELISA test results available for analysis in the combined two-year test period (Table 1). Positive percent agreement of rapid tests versus ELISA was 88.5% (95% Confidence Interval [CI]: 85.7-91.3%). At the regional level, PPA was 83.4% (CI: 77.9-88.9%) in the south, 89.7% (CI: 85.4-94.0%) in the center, and 89.2% (CI: 85.0-93.4%) in the north of the country. The median PPA across all 36 surveillance sites was 88.8% (Interquartile Range [IQR]: 83.1-93.0%, minimum 63.8%, maximum 100.0%); Nineteen sites had a PPA below 90% (See Additional file 1: Table S1, which shows site-level measures of agreement). However, when analyzed by survey year, the pooled PPA was found to increase from 86.5% (CI: 82.5-90.4%) in 2007 to 90.6% (CI: 87.6-93.5%) in 2009 (p = 0.040). Negative percent agreement for both years combined was 98.9% (CI: 98.5-99.2%), and above 98% in all three regions and in both years. The median NPA across the surveillance sites was 99.1% (IQR: 98.6-99.5%). No significant difference was found among the three regions for either PPA (p = 0.203) or NPA (p = 0.210), but as was the case with PPA, NPA was significantly higher in 2009 than in 2007 (p = 0.028) (Table 2).

**Table 1:** Availability of and differences between routine PMTCT and ANC surveillance test results in Mozambique

		**Availability of routine PMTCT HIV status for specimens with ANC surveillance results**									
	**n**	**Routine PMTCT HIV status not available**				**% missing routine PMTCT HIV status**	**Total with routine PMTCT and ANC surveillance tests**	**Comparison between routine PMTCT (R) and ANC surveillance (S)**			
		**Total**	**Refused PMTCT HIV test**	**Prior positive HIV test**	**Other reason**^**1**^			**R-S+**	**R+S+**	**R+S-**	**R-S-**
Region											
South	5891	162	16	23	123	2.75	5729	128	1058	71	4472
Center	8895	220	2	39	179	2.47	8675	147	1278	69	7181
North	6436	277	65	3	209	4.30	6159	84	422	59	5594
Year											
2007	10588	224	82	9	133	2.12	10364	213	1359	125	8667
2009	10634	435	1	56	378	4.09	10199	146	1399	74	8580
Total	21222	659	83	65	511	3.11	20563	359	2758	199	17247

Notes:

1. Refers to missing data in the ANC surveillance register with no documented refusal or prior positive HIV test.

**Table 2:** Percent agreement of routine PMTCT HIV status versus ANC surveillance test results in Mozambique

	**n**	**Positive percent agreement (95% CI)**	**Negative percent agreement (95% CI)**
Region			
South	5729	83.4 (77.9-88.9)	99.0 (98.5-99.4)
Center	8675	89.7 (85.4-94.0)	99.0 (98.6-99.5)
North	6159	89.2 (85.0-93.4)	98.4 (97.7-99.1)
Year			
2007	10364	86.5 (82.5-90.4)	98.6 (98.1-99.0)
2009	10199	90.6 (87.6-93.5)	99.1 (98.8-99.5)
Total	20563	88.5 (85.7-91.3)	98.9 (98.5-99.2)
Median (IQR)	36	88.8 (83.1-93.0)	99.1 (98.6-99.5)

Notes:

1. Positive percent agreement calculated as R+S+/(R+S+ and R-S+) where R+/R- refers to routine PMTCT HIV status and S+/S- refers to ANC surveillance HIV status.

2. Negative percent agreement calculated as R-S-/(R+S- and R-S-).

The median of the 36 HIV site-level prevalence estimates calculated from routine PMTCT results was 13.5% (IQR 7.0-21.4%), while the median of the 36 HIV site-level prevalence estimates calculated from surveillance data was 14.4% (IQR 8.2-21.8%). When HIV test results were pooled across sites, HIV prevalence from routine PMTCT data was 14.4% versus 15.2% from surveillance data. In absolute terms, HIV prevalence was 0.78 percentage points lower when measured from routine PMTCT rapid tests, representing a 5.1% relative decrease in prevalence compared with the results of ELISA tests performed on matched specimens from the same participants (Table 3). The relative difference in HIV prevalence at the level of each surveillance site varied from 0-22.2%, after excluding one outlier of 100%, and the absolute difference varied from 0-4.3 percentage points (See Additional file 2: Table S2, which shows site-level difference in prevalence between routine PMTCT and ANC surveillance test results). As a surveillance system based on PMTCT test results would likely classify previously-diagnosed women as HIV-positive, we also re-calculated the PMTCT prevalence estimate including known positives. Given the small number of women who refused a PMTCT test due to known HIV status (n = 65, Table 1) the potential impact of including these women in the HIV prevalence estimate derived from routine PMTCT data was minimal (14.5% or 0.77 percentage points lower versus 0.78 percentage points lower when excluded, calculations not shown). Two of these 65 “known positives” had a negative ELISA test result.

**Table 3:** Differences in HIV prevalence between routine PMTCT HIV status and ANC surveillance tests in Mozambique

	**n**	**Prevalence (%)**		**Difference in prevalence**	
		**ANC surveillance**	**Routine PMTCT**	**Relative**^**1**^	**Absolute**^**2**^
Region					
South	5729	20.70	19.71	−4.8	−1.00
Center	8675	16.43	15.53	−5.5	−0.90
North	6159	8.22	7.81	−4.9	−0.41
Year					
2007	10364	15.17	14.32	−5.6	−0.85
2009	10199	15.15	14.44	−4.7	−0.71
Total	20563	15.16	14.38	−5.1	−0.78

Notes:

1. relative difference between routine PMTCT HIV status (R) and ANC surveillance (S) calculated as (R-S)/S where R+/R- refers to routine PMTCT HIV status and S+/S- refers to ANC surveillance HIV status.

2. absolute difference between routine PMTCT HIV status (R) and ANC surveillance (S) calculated as (R-S).

Pooled HIV prevalence based on ANC surveillance among those who refused PMTCT testing for reasons other than a previous diagnosis (n = 83) was 10.8% (CI: 6.6-15.0%), compared with 15.5% (CI: 12.8-18.2%) among non-refusers. Among those women missing PMTCT results for other reasons (n = 511), prevalence was 18.4% (CI: 12.5-24.3%). The ratio of HIV prevalence among refusers compared with non-refusers was 0.70, while the ratio of HIV prevalence among women missing routine PMTCT results for reasons other than refusal with those not missing results was 1.19. The percent bias that would be introduced by excluding those who refused routine PMTCT testing from the sample was 0.12%, while the percent bias that would be introduced by excluding those who had missing routine PMTCT test results for reasons other than refusal from the sample was -0.47% (Table 4).

**Table 4:** Potential effect of missing routine PMTCT HIV status on estimated HIV prevalence in Mozambique

***A. ANC surveillance HIV prevalence by routine PMTCT HIV test status***					
	**Total**	**ANC test results by availability of PMTCT rapid test**			
		**Due to refusal**		**Due to reasons other than refusal or known positive**	
		**PMTCT HIV status missing due to refusal**	**PMTCT HIV status not missing due to refusal**	**PMTCT HIV status missing due to other reasons**	**PMTCT HIV status not missing due to other reasons**
HIV+	3283	9	3274	94	3189
Total	21222	83	21139	511	20711
HIV Prevalence	15.5%	10.8% (6.6-15.0)	15.5% (12.8-18.2)	18.4% (12.5-24.3)	15.4% (12.7-18.1)
***B. Measures of effect of missing test results on HIV prevalence***					
**Measure**		**Due to refusal**		**Due to reasons other than refusal or known positive**	
Absolute difference^1^		−4.6%		3.0%	
Relative difference^2^		−30.0%		19.5%	
Percent bias^3^		0.12%		−0.47%	
Prevalence ratio^4^		0.70		1.19	

Notes:

1. Absolute difference is the HIV prevalence in those whose PMTCT HIV status is missing minus the HIV prevalence in those for whom it is present.

2. Relative difference is the absolute difference divided by the HIV prevalence among those for whom the PMTCT HIV status is present.

3. Percent bias is the HIV prevalence in those for whom PMTCT HIV status is present minus the overall HIV prevalence, divided by the overall HIV prevalence.

4. The prevalence ratio is the prevalence amongst those for whom the PMTCT HIV status is missing divided by the prevalence among those for whom the PMTCT HIV status is not missing.

## Discussion

There is increasing interest in the use of routine PMTCT data to supplement or replace ANC surveillance surveys [13]. Countries and agencies such as WHO and the U.S. Centers for Disease Control and Prevention have begun looking at the availability of routine PMTCT data, their quality, differences in populations covered by PMTCT testing and ANC surveillance, as well as issues related to differences in diagnostics. Finally, investigators have calculated the likely impact of these differences on HIV prevalence estimates [14-17]. In spite of varying levels of availability and quality of routine PMTCT data, and less-than-universal uptake of PMTCT, prevalence estimates are often robust at the national level yet variable at the site level and investigators have generally been cautious about recommending a switch to routine data sources for ANC surveillance purposes [13]. This may be due in part to the use in many countries with generalized HIV epidemics of modeling tools such as Spectrum which rely on site-level HIV prevalence data to estimate national HIV prevalence trends [18] and which may be sensitive to differences in site-level estimates.

Switching to routine sources for ANC surveillance purposes represents a potential long term cost savings for national HIV programs. Such savings will not only depend on system design but also on the cost of improving routine PMTCT HIV testing and data collection systems to the point where they can be used for surveillance. The degree to which such improvements will strengthen the health system will depend on whether they will affect the entire program or just a limited number of sentinel sites.

Many evaluations of the suitability of routine HIV testing for surveillance purposes have focused on bias due to refusals, while few, if any, have looked at differences that may arise due to testing methods. This evaluation focused specifically on issues of consistency of test results in a matched sample of PMTCT rapid test and centralized ELISA test results and the impact that differences in test results and uptake of rapid PMTCT HIV testing would have on prevalence estimates. Studies designed to assess diagnostic performance rely on the existence of a pre-existing reference standard to define true positives and true negatives [6]. We found relatively low levels of positive percent agreement for the PMTCT HIV rapid test results in this study, given the high sensitivity and specificity of modern HIV tests regardless of ELISA or rapid format. Based on published operational characteristics of the tests used in this study, the expected net sensitivity and specificity of both the rapid test and ELISA algorithms are 100%. This inconsistency between the positive percent agreement and the published operational characteristics of the tests implies that either the ELISA or rapid tests are not performing as published or test results are not being documented correctly. Future studies would need to be undertaken to determine to what degree these inconsistencies can be attributed to rapid tests, ELISA tests, or documentation errors.

In spite of the low levels of agreement found in this study, the impact on prevalence estimates among pregnant women was modest: HIV prevalence estimates based on PMTCT rapid test results at ANC surveillance sites would have been less than one percentage point lower than those obtained from centralized ELISA testing, although relative differences ranged from 0-22.2% at the site level. The overall difference of less than one percentage point among pregnant women in Mozambique would be unlikely to trigger a change in national HIV policy. However, as ANC estimates may be the most reliable HIV prevalence data available in districts where sentinel sites exist, site-level differences could influence local interpretations of the status or course of the HIV epidemic.

The impact of differential refusal bias was also quite limited: although the relative difference in HIV prevalence was lower in refusers compared to non-refusers (10.8% vs. 15.5%), the low PMTCT test refusal rate of only 0.4% means HIV estimates would only have been increased by 0.12 percentage points if results from those who refused HIV testing during PMTCT were removed. Although there were only 83 refusers in this study, there were an additional 511 women who were missing rapid test results but who did not have a documented refusal or history of a positive HIV test. Interestingly, HIV prevalence in these women was higher than those who were not missing results for reasons other than refusal or known status (18.4% vs. 15.4%). It is possible this group included women whose known status was not reported or not correctly recorded. However, had these women been excluded from the ANC surveillance system prevalence would have only decreased by 0.47 percentage points.

Given the near-universal uptake of both PMTCT testing and ANC surveillance, participation biases are not a primary concern for migrating to routine data sources for ANC surveillance in Mozambique. However, the poor positive percent agreement between rapid and ELISA-based testing is concerning. There are various possible explanations, including: 1) problems with test manufacturing or storage, 2) problems with specimen storage or handling, 3) non-compliance with recommended rapid and/or ELISA testing procedures, such as not waiting for the designated period before interpreting rapid test results, 4) data entry or transcription errors during the ANC visit or when entering surveillance data centrally, 5) swapping of test results between specimens due to unlinked anonymous testing or for other reasons, 6) differing window periods which could lead to biological false-negatives. Double entry of data and automatic transcription of laboratory test results, and internal quality assurance procedures for ELISA-based testing reduced the likelihood of some, but not all types of errors. External quality assurance has generally found low (≤1%) discrepancy rates for ANC surveillance testing. The national HIV external quality assessment program was established in 2006 by the National Institute of Health to monitor the quality of diagnostic HIV rapid testing. The program includes proficiency testing and included ANC nurses from PMTCT programs at 11 of the 36 sentinel sites in 2007 and 13 of 36 sites in 2009.

The lack of a reference standard to assign the true HIV status of each participant, along with our inability to identify the relative contribution of each source to the differences between PMTCT testing and ANC surveillance are the primary limitations of this study. Nonetheless, these limitations do not diminish the value of being able to identify these discrepancies, assess their impact on HIV prevalence estimates at the aggregate and site levels, and assess the potential impact of refusal bias on prevalence estimates.

While this study focused on HIV prevalence estimates among pregnant women at the national and regional levels, ANC surveillance data are routinely used to assess the impact of the Mozambican HIV epidemic through mathematical models including Spectrum that rely on site-level prevalence estimates, thus a sensitivity analysis that assesses the impact of the use of routine PMTCT data in these models should be performed before recommending a switch to routine PMTCT test data for surveillance purposes.

Although switching to PMTCT-based surveillance would be premature in Mozambique, comparisons between PMTCT and ANC surveillance results should be repeated in the future to verify the potential improving trend in agreement seen from 2007-2009. A more comprehensive evaluation of routine PMTCT HIV test results should be conducted at ANC surveillance sites in order to determine if data will be sufficiently available and complete to allow their use for surveillance. Furthermore, the national HIV external quality assessment program should be expanded to cover service delivery points at all sites which may participate in a future surveillance program based on routine HIV tests, including all existing ANC sentinel surveillance sites. Sites which reported PPA less than 90% should be evaluated closely to determine, if possible, the underlying causes of poor test result agreement. Establishing a comprehensive quality management program at ANC surveillance sites could further reduce discrepancies that are due to site-level issues with HIV diagnostic testing.

## Conclusions

The poor agreement across test results found in this study implies that tests are not performing consistent with their published operational characteristics and indicates it would be premature to replace ANC surveillance with routine PMTCT test data. However, the significant improvements in agreement seen from 2007 to 2009, should they continue in the future, are cause for optimism. In addition to confirming this possible positive trend in agreement rates and assessing the sensitivity of the Spectrum model to variation in site prevalence estimates, it will also be important to evaluate the availability, quality and completeness of HIV test results originating from routine ANC data systems before recommending that these data be used to replace data collected in ANC surveillance surveys.

## Abbreviations

CDC: U.S. Centers for Disease Control and Prevention;DBS: Dried blood spot;ELISA: Enzyme-linked immunosorbent assay;IQR: Inter-quartile range;NPA: Negative percent agreement;PMTCT: Prevention of mother-to-child transmission;PPA: Positive percent agreement;RPR: Rapid plasma reagin

## Competing interests

The authors declare that no competing interests exist.

## Authors’ contributions

PWY led manuscript development, PWY, RZH and MM conducted the analyses, MM oversaw data and specimen collection, RWS assisted with data analysis and interpretation of results, IVJ assisted with interpretation of results. All authors read and approved the final manuscript.

## Pre-publication history

The pre-publication history for this paper can be accessed here:

http://www.biomedcentral.com/1471-2334/13/96/prepub

## Supplementary Material

Additional file 1: Table S1 Site-level measures of agreement between routine PMTCT and ANC surveillance test results in Mozambique. A table in XLS format showing positive and negative percent agreement of routine PMTCT HIV status by sentinel site in pregnant women in Mozambique, 2007-2009. Click here for file

Additional file 2: Table S2 Site-level difference in HIV prevalence between routine PMTCT and ANC surveillance test results in Mozambique. A table in XLS format showing availability of routine PMTCT test results for specimens with ANC surveillance test results, and differences in test results and HIV prevalence by sentinel site in pregnant women in Mozambique, 2007-2009. Click here for file
